# Milking the Alternatives: Understanding Coffee Consumers’ Preferences for Non-Dairy Milk

**DOI:** 10.3390/bs14070569

**Published:** 2024-07-05

**Authors:** Nibal Halabi, Velina Hristova, Ivo Vlaev

**Affiliations:** 1 Warwick Business School, University of Warwick, Scarman Rd., Coventry CV4 7 AL, UK; nibal.halabi@warwick.ac.uk; 2 Psychology Department, Sofia University “St. Kliment Ohridski”, bul. “Tsar Osvoboditel” 15, 1504 Sofia, Bulgaria; 3 Institute for Population and Human Studies, Bulgarian Academy of Sciences, Acad. G. Bonchev Str. 6, 1113 Sofia, Bulgaria

**Keywords:** consumer behavior, Theoretical Domains Framework, Behavior Change Wheel, non-dairy industry, plant-based dairy alternatives

## Abstract

Consumer interest in plant-based milk alternatives is growing, despite extra charges in coffeehouses. While much research exists on non-dairy alternatives, plant-based milks in coffee drinks remain understudied. This study examines consumer preferences and behaviors regarding milk alternatives in coffee, using the Theoretical Domains Framework (TDF) and the Behavior Change Wheel (BCW). A survey of 200 participants from 19 countries explored demographics, coffee habits, attitudes towards non-dairy milk charges, and marketing awareness. Market insights showed taste as the main reason for coffee choice, with a preference for cow’s milk and local cafes for quality. Many opposed the extra charges for non-dairy options, citing lactose intolerance or allergies, especially among Gen Z and Millennials. Regional variations included stronger opposition in the UK and Germany compared to the UAE and USA. The marketing for non-dairy milk was less memorable than general coffee advertisements. Regression analysis confirmed that viewing non-dairy milk as a dietary staple increased consumption, aligning with the TDF’s “Beliefs about Consequences” domain. Finally, within the BCW framework, the intervention strategies centered on training and coercion were discussed. Implementing these approaches could encourage the wider adoption of non-dairy milk options in coffee shops, fostering inclusivity, health awareness, and supporting environmental sustainability efforts.

## 1. Introduction

Increasing interest in sustainability, along with concerns for the environment and animal welfare, is motivating more consumers to choose plant-based milks. The marketing of plant-based dairy alternatives (PBDA) often emphasizes their commitment to sustainability, environmental protection, natural ingredients, and humane animal treatment, which gives them a competitive edge over traditional dairy products.

Across key environmental metrics—such as land use, greenhouse gas emissions, water use, and eutrophication—cow’s milk has a much greater impact, producing about three times more greenhouse gases [1]. In comparison, research indicates that plant-based drinks have significantly lower greenhouse gas emissions, reduced by 59–71% per 250 mL [2]. Dairy production also uses around ten times more land, consumes two to twenty times more freshwater, and contributes significantly more to eutrophication [1]. Among plant-based options, almond milk has lower greenhouse gas emissions and land use than soy milk but requires more water and results in more eutrophication. While all plant-based alternatives are more environmentally friendly than dairy, none is the clear winner across all sustainability metrics. Oat and soy drinks, for instance, use considerably less water than dairy milk, yet rice drinks do not share this advantage. However, consumers must navigate these nuances to make informed and sustainable choices, as each type of plant-based milk presents its own set of environmental trade-offs.

Additionally, reducing our reliance on animal-based foods aligns with several United Nations Sustainable Development Goals (UN SDGs), particularly those focused on sustainable consumption and production, the sustainable management of natural resources, and urgent climate action [3]. By adopting planetary diets, which prioritize plant-based foods, individuals can significantly decrease their environmental footprint, helping to mitigate climate change, preserve biodiversity, and promote the sustainable use of resources. Research shows that substituting the 250 mL serving of dairy milk allowed within the EAT Lancet Planetary Health Diet with a fortified plant-based alternative maintains nutritional adequacy while substantially lowering environmental impact [4,5].

### 1.1. The Individuals’ Health Factor

The most common adverse reactions associated with milk consumption are cow’s milk protein allergies (CMPAs) and lactose intolerance (LI). These conditions arise from the body’s inability to properly digest, absorb, and metabolize specific components of milk. In the case of CMPA, the immune system reacts abnormally to proteins found in cow’s milk, leading to allergic reactions. Lactose intolerance, on the other hand, is due to the malabsorption of lactose, the sugar in milk, caused by a deficiency of the enzyme lactase. This deficiency is particularly prevalent, with about two-thirds of the world’s population affected, though the prevalence varies significantly across different regions [6].

Digestibility issues and lactose intolerance are the primary reasons for consumers to turn to plant-based milks [7,8,9]. For those with LI, consuming dairy can lead to symptoms like bloating, diarrhea, and stomach cramps, making dairy alternatives a more comfortable choice. Similarly, individuals with CMPA must avoid cow’s milk altogether, necessitating alternatives that do not trigger allergic reactions.

These issues have compelled specific populations to seek out milk alternatives that are nutritionally similar to conventional milk.

### 1.2. Nutritional Content of Dairy versus Plant-Based Beverages

Studies from different parts of the world have analyzed the nutritional content of non-dairy milk compared to plant-based alternatives [2,10,11,12,13,14,15]. Plant-based milk alternatives (PBMAs) have distinct nutritional profiles compared to bovine milk, influenced by their plant sources, processing methods, and fortification with additional ingredients.

Cow and goat milks are naturally rich in essential minerals such as calcium, potassium, magnesium, sodium, and phosphorus, as well as trace elements like selenium and zinc [10]. Conversely, PBMAs require fortification to reach similar nutritional levels. Furthermore, PBMAs contain antinutrients like phytic acid, trypsin inhibitors, and inositol phosphates, which can interfere with mineral absorption and reduce protein digestibility but also provide beneficial components that are not present in cow’s milk, such as isoflavones (especially in soy-based alternatives) and dietary fibers [9].

Regarding macronutrients, PBMAs typically have a lower protein content than cow’s milk, with animal proteins being superior in nutritional quality and digestibility, due to their varied amino acids [16]. Only soy-based milk has a protein content comparable to that of cow’s milk [13].

Unlike cow’s milk, PBMAs are free from lactose and cholesterol and have higher levels of unsaturated fatty acids. Additionally, PBMAs have very low levels of saturated fats, minimal sodium [4], and have no cholesterol, all of which contribute positively to a healthier diet. Additionally, dairy consumption is related to some dermatological conditions [9,17].

### 1.3. Pricing Strategies in the Dairy versus Alternative Milk Industry

Shareholders and common ownership significantly influence the dynamics of the dairy and alternative milk industry. Institutional investors often hold stakes in multiple companies within these sectors, driving pricing decisions to maximize portfolio returns. When non-dairy milk producers are commonly owned by the same institutional investors, competitive pricing incentives may diminish, potentially leading to higher prices for non-dairy milks.

An OECD document from 2017 notes the rise in common ownership, with institutional investors holding stakes in multiple-sector companies [18]. Alex Edmans discussed, in a 2009 article, how shareholder activities impact market dynamics and corporate governance, including pricing strategies [19]. Aghion et al. [20] also explored how liquidity impacts shareholder incentives, noting that easily tradable shares can lead to short-term profit strategies at the expense of long-term company health. In the context of pricing, this managerial myopia might result in higher prices and underinvestment in necessary innovations, explaining the high cost of non-dairy products. While coordinated pricing among commonly owned firms can lead to stable prices, it might also reduce the competitive pressure, keeping prices high, which negatively impacts price-sensitive consumers or those dependent on non-dairy milks due to dietary restrictions. The OECD suggests that common ownership may encourage unilateral pricing behaviors that favor institutional investor portfolios, potentially facilitating collusive outcomes [18]. This can lead to pricing strategies, such as charging extra for non-dairy milks, that prioritize short-term profits over consumer interests and market sustainability.

Unless someone has a health issue or concern, such as diet consciousness, lactose intolerance, or a dairy allergy, there is one large barrier to entry for the adoption and proliferation of plant-based milks: cost. One of the primary challenges facing PBMAs is their elevated production costs, which impact every stage of the plant-based milk supply chain [9]. The higher production costs of plant milk (due to expensive processes, packaging, marketing, and logistics) compared to dairy milk provide plant milk suppliers with significant bargaining power when negotiating with retailers like large coffee shops [21]. While retailers face a medium level of buyer bargaining power and some flexibility in choosing suppliers, partnering with suppliers can reduce costs through larger purchase quantities and optimized supply chain management.

**Figure 1:**
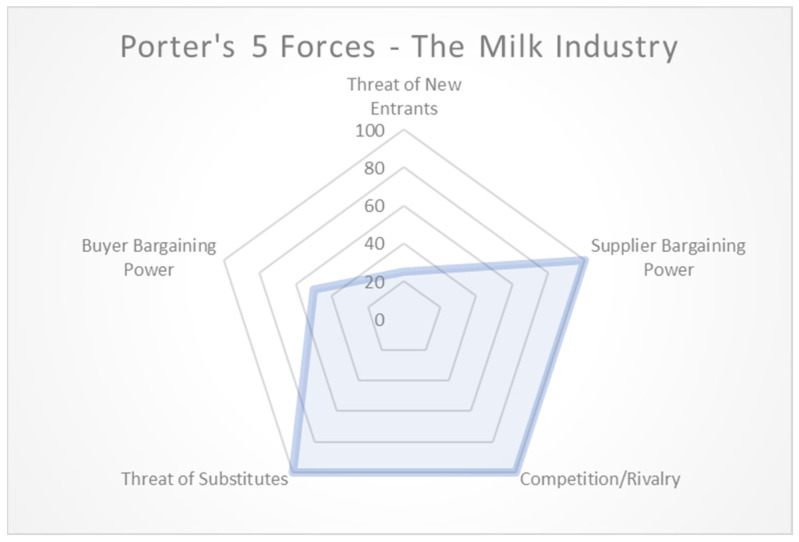
Porter’s Five Forces—dairy and plant-based milks.

Once retailers acquire soy milk and offer it to customers, their bargaining power remains constrained—they cannot provide the product for free, yet customers can choose alternative suppliers where costs are lower or the product is free. This complex market landscape, including various dairy milk alternatives and consumer preferences like drinking espresso or coffee without milk, illustrates the intricate dynamics described by Porter’s Five Forces model, as depicted in Figure 1.

While there is substantial research on non-dairy alternatives, there is a notable lack of studies specifically focusing on the use of plant-based milks in coffee drinks. This study aims to fill this gap by examining consumer preferences and behaviors related to non-dairy milk alternatives in the context of coffee consumption.

### 1.4. Theoretical Domains Framework (TDF) Model

To achieve its goal, the present study employed the Theoretical Domains Framework (TDF) [22] within the Behavior Change Wheel (BCW) [23] approach. The TDF was chosen because it offers a comprehensive framework that integrates 128 constructs from 33 behavior change theories into 14 domains, providing a thorough understanding of the factors influencing behavior, including the following: knowledge, skill, beliefs about capabilities, beliefs about consequences, behavioral regulation, optimism, emotion, environmental context and resources, social/professional role and identity, intention, goal, reinforcement, social influence, and memory, attention, and decisional processes. Secondly, the BCW complements the TDF by offering a structured approach to designing and evaluating behavior change interventions, ensuring that this study’s findings can be effectively translated into practical strategies for promoting non-dairy milk consumption. By combining these frameworks, this study can uncover a nuanced picture of consumer behaviors and preferences, identify key barriers and facilitators, and develop targeted interventions to encourage the adoption of non-dairy milk alternatives in coffee shops.

The TDF has demonstrated its effectiveness and utility within public health and healthcare contexts, dietary behaviors [22,24,25], the promotion of children’s milk consumption [26], and the adoption of sustainable agri-environmental practices [27].

However, the utilization of this behavior change methodology is not widely embraced within the domain of coffee consumer choice behavior, especially dairy alternatives. Therefore, to our knowledge, this is the first study to use the TDF and other components of the BCW to explore the barriers and facilitators in the context of non-dairy and plant-based milks in coffee drinks. Previous research has investigated barriers to the consumption of plant-based beverages among US consumers using a multi-response method (emotional, conceptual, situational, and conative) [28]. It was found that fitting expectations and negative associations were dominant among consumers who had never consumed plant-based beverages, with these associations varying by product type. For instance, negative perceptions were the strongest for oat milk, followed by fruit smoothies with soy milk, and iced coffee with almond milk. Food neophobia also negatively influenced the perceptions of less familiar products, including plant-based beverages. However, this research was limited to a small number of beverages and plant-based alternatives. Another systematic review investigated the determinants of real-life behavioral interventions to promote more plant-based and less animal-based diets, finding that targeting individual or environmental determinants is most effective [29]. None of these studies used the TDF and BCW, nor did they investigate plant-based alternatives to dairy specifically for coffee drinks.

## 2. Materials and Methods

### 2.1. Subjects and Design

This study employed a cross-sectional design. A total of 215 participants from 19 countries, including prominent regions such as the UAE, USA, UK, Germany, and Canada, participated in this study. However, 15 participants were excluded due to incomplete survey submissions, ensuring the integrity of our results. The final sample size consisted of 200 respondents. Of these 200 participants, approximately 56% identified as male, 42% as female, and 4 participants (2%) identified as transgender, non-binary, or another not-listed gender. According to the Beresford Research classification of age groups, Millennials (aged 26–41) constituted the largest demographic in this study, comprising 53% (106 participants) of the sample [30]. Generation X (aged 42–57) accounted for 26% of respondents, while Generation Z (aged 18–25) made up 16%. Geographically, roughly one quarter of the respondents (23%) resided in the USA, followed by 22% in the United Arab Emirates. The UK and Germany each accounted for around 11% of participants, and Canada represented 9%. The participants were also asked about food allergies, with 81% indicating they do not have any, while about 10% of those with an allergy reported having a milk allergy.

### 2.2. Instruments

The survey utilized in this study consisted of 31 questions, including a 10-item TDF-based scale. The survey began with a series of 7 questions to capture the demographic information about the participants. Following the inquiry about coffee consumption, the participants who reported being coffee drinkers proceeded to respond to nine more questions related to coffee, whereas non-coffee drinkers moved directly to the next section of the survey. Following the coffee-related questions, all 200 participants answered 3 questions related to their attitudes towards non-dairy milk charges at coffee shops. After this, the participants responded to two questions about their awareness of marketing campaigns for coffee or coffee shops in general, as well as specific promotions for non-dairy milk coffee beverages.

The final section of the survey asked the participants to evaluate 10 statements developed using the Theoretical Domains Framework (TDF). The survey instrument was developed specifically for this research by the authors and had not been previously validated. It was constructed based on elements and recommendations from the Theoretical Domains Framework (TDF) [23], informed by previous TDF surveys and interview guides [31,32,33], as well as a validated TDF scale [34]. The full questionnaire is provided in Appendix A.

### 2.3. Procedure

Primary data were collected online through a survey featuring both closed and open-ended questions. The survey was administered using a Computer-Assisted Web Interviewing (CAWI) platform, with data collection occurring from May to July 2023.

The survey was reviewed and approved by Warwick Business School (WBS), following the guidelines of the University of Warwick’s Humanities and Social Science Research Ethics Committee (HSSREC) on 15 July 2022. All participants provided informed consent before participating. The survey was conducted anonymously, adhering to GDPR compliance, as per the University of Warwick’s data protection policies. Detailed information on data management practices is available in the University of Warwick Research Privacy Notice.

### 2.4. Data Analysis

To comprehensively explore the participants’ coffee-related behaviors and preferences and gain market research insights, a series of analytical methods were employed in this study. A frequency analysis was conducted to identify patterns and distributions in the participants’ coffee-related behaviors, attitudes, and preferences. This analysis involved examining the frequencies of responses to questions regarding coffee consumption habits, preferences for types of milk, and locations where coffee is typically consumed. The results of this analysis provided valuable insights into the prevalence of various behaviors and preferences among the study participants, offering a picture of their coffee consumption patterns. In addition to exploring the behaviors and preferences, this study conducted market research comparisons based on geographical location, age group, attitudes towards charges for non-dairy milks, and awareness of marketing campaigns. Multiple regression analysis was performed to investigate the relationship between the Theoretical Domains Framework (TDF) questions (independent variables) and the consumption of non-dairy milk (dependent variable). This analysis provided a deeper understanding of which TDF domains significantly influenced the participants’ likelihood of adopting or rejecting non-dairy milk. Finally, based on the BCW, practical suggestions for intervention strategies were discussed.

## 3. Results

### 3.1. Coffee-Related Behavior

Of the 200 participants, 177 (approximately 89%) were coffee drinkers and responded to the coffee-related questions. The first question inquired why the participants drink coffee, offering four predefined options and an “other” category, with the participants being allowed to select multiple reasons. The most common reason was taste, selected by about 85% of the respondents, followed by caffeine or energy at 64%. Social reasons accounted for 47%, stress relief for 26%, and 10% chose “other.”

Understanding why consumers drink coffee is essential for grasping their preferences for different types of coffee, such as milk-based or black. The subsequent five questions explored the participants’ coffee drinking habits, including the time of day and preferred locations, providing insights into their consumption patterns. The data revealed that 92% of the respondents primarily drink coffee at home, followed by 64% at coffee shops, and 58% at the office. These insights highlight the various factors influencing coffee drinkers’ preferences. For instance, those drinking coffee at the office might face challenges with dairy-alternative milks due to limited company provisions, whereas coffee shops offer a wider range of options.

The participants were then asked about their milk preferences in coffee. The responses indicated that 61% preferred cow’s milk, 53% chose no milk or espresso, and 51% opted for plant-based or other milks. This question is significant for understanding the milk preferences of coffee drinkers and serves as a key reference point for our later analysis.

The final two questions asked the coffee drinkers about their preferred coffee shop or brand and the reasons for their preference. Among the 177 respondents, approximately 40% (69 individuals) preferred local cafes or small businesses, 15% favored Starbucks, 10% chose another large coffee chain or brand, and 30% selected home or other options. The most frequently cited reasons for their preferences were the quality or taste of the coffee (83%), followed by the ambiance (37%), and the location or availability (36%).

### 3.2. Attitudes towards Non-Dairy Milk Charges at Coffee Shops

After completing the coffee section of the survey, all 200 participants were asked three questions about dairy milk alternatives and their preferences. The first question assessed their awareness of the additional charges for non-dairy milks at coffee shops. More than half (54%) indicated that their coffee shop charges extra, 14% said that it does not, and 32% were unsure or unaware of the policy.

**Figure 2:**
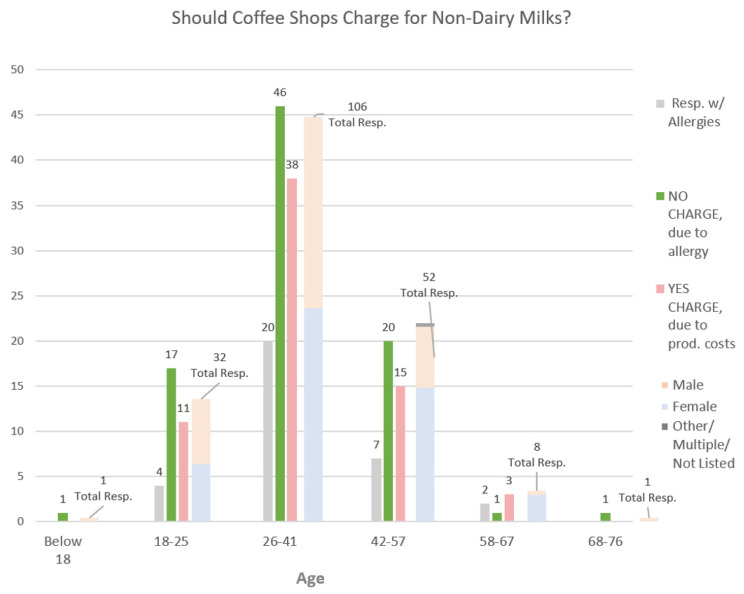
Distribution of data by participants’ beliefs on whether customers should pay extra for non-dairy milks.

The second question explored the participants’ beliefs on whether customers should pay extra for non-dairy milks (Figure 2). Among the “yes” responses, the most common reason was that non-dairy milk costs more to produce (34%). The highest response overall was “No, because customers should not have to pay due to an allergy/intolerance” (43%).

When analyzed by age group, Gen Z (18–25) most strongly believed that non-dairy milk should not be chargeable due to allergies (53%), followed by Millennials (26–41) at 43%, Gen X (42–57) at 39%, and Boomer I (58–67) at 13%. The youngest and eldest groups, each with one respondent, both scored 100%, which were considered statistical anomalies.

The final question related to non-dairy milk charges asked the participants if they would visit a coffee shop or brand more often if it did not charge extra for non-dairy milks. Nearly 43% stated that this would not change their behavior, with about 22% more saying that they do not drink dairy milk, so it does not affect them. The remaining responses were affirmative, with 15% saying they would always visit or order from such a coffee shop more often, and 21% mentioning they would do so only if the shop was close by or convenient.

### 3.3. Marketing Campaigns Awareness

After completing the survey section on dairy milk alternatives, the participants were asked about their awareness of marketing campaigns for coffee and specifically for non-dairy milk coffee beverages. Notably, 61% of the respondents recalled seeing advertisements for a coffee shop or brand, while 35% remembered campaigns promoting non-dairy milk beverages.

### 3.4. TDF Framework Results

The participants’ perceptions regarding dairy and non-dairy milk were further assessed through 10 TDF-based items. The ten TDF statements, along with their scores, means, standard deviations, and variances, are detailed in the table below (Table 1).

The statement regarding taste yielded a mean response of 2.65, indicating a slight negativity towards non-dairy milk taste, although the high standard deviation (1.28) highlighted diverse opinions. Social influences were explored in the statement about peers and family members not consuming non-dairy milk, which had a mean of 2.59, indicating variability. The wording may have influenced the responses, necessitating cautious interpretation. Concerns about the cost showed a mean response of 3.35, suggesting a generally affirmative perception with a narrower range of opinions. The participants strongly agreed (mean 4.47) with the statements related to dairy consumption in upbringing, indicating a widespread consensus. Statements on health considerations generally reflected positive inclinations towards incorporating non-dairy milk into diets, with means of around 3.16 and relatively consistent responses, underscoring growing health awareness and dietary flexibility among the participants.

### 3.5. Regression Analysis Results

The regression analysis explored how the TDF questions (independent variables) relate to non-dairy milk consumption (dependent variable) (Table 2). The results showed a moderate positive correlation (Multiple R = 0.5969), indicating that higher agreement with the TDF questions was associated with increased non-dairy milk consumption About 35.63% of consumption variance was explained by TDF question variations (R Square = 0.356). The model was statistically significant (F = 10.46, *p* < 0.001), confirming that TDF questions collectively influenced consumption. Notably, questions on dairy and non-dairy milk in diets had the lowest *p*-values, indicating a significant impact on consumption.

The coefficient for “Dairy milk as part of a normal diet” was −0.072, indicating a negative association with non-dairy milk consumption (Table 2). In contrast, “Non-dairy milk as part of a normal diet” had a coefficient of 0.066, signifying a positive association—suggesting that those who view non-dairy milk as a dietary staple are more inclined to consume it. Conversely, other factors such as taste, price, availability, social influence, and calorie consciousness did not exhibit statistically significant correlations with non-dairy milk consumption based on our analyses.

### 3.6. Geographical Location

**Figure 3:**
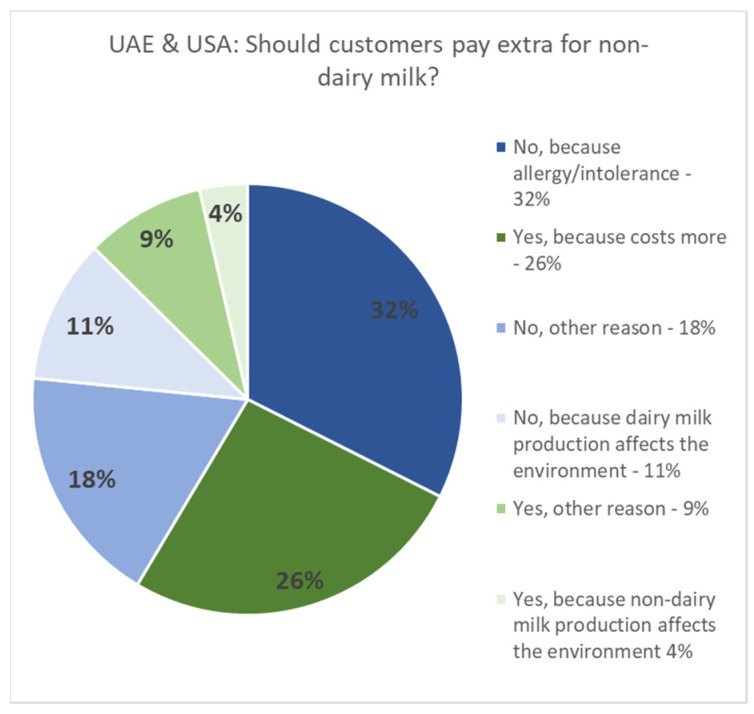
Distribution of data by participants’ beliefs on whether customers should pay extra for non-dairy milks based on geographical location: UAE & USA.

**Figure 4:**
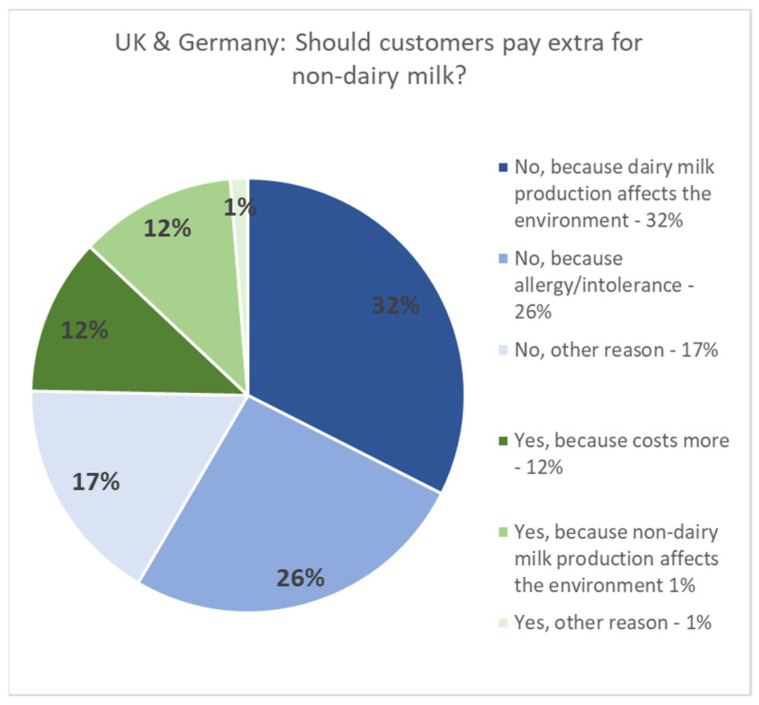
Distribution of data by participants’ beliefs on whether customers should pay extra for non-dairy milks based on geographical location: UK & Germany.

Of particular interest is the high representation of participants from the United States and the United Arab Emirates, where Starbucks imposes a “plant-based tax” by charging extra for non-dairy milk. In contrast, the survey also identified the United Kingdom and Germany as the third and fourth most represented countries, respectively, where Starbucks does not charge for non-dairy milks [35]. The strong representation from these four countries suggests that their perspectives, behaviors, and opinions may have a significant influence on the overall findings, as visualized in Figure 3 and Figure 4 below.

A significant majority of respondents from the UK and Germany across all age groups believe that no one should pay additional charges for non-dairy milk, regardless of the circumstances, compared to respondents from the UAE and USA. Although the younger respondents from the UAE and USA also prioritize inclusivity similarly to their counterparts in the UK and Germany, their consideration appears to be balanced with economic considerations favoring the USA and UAE respondents.

Based on awareness of marketing campaigns (Table 3), Canada had the highest visibility for coffee advertisements (82%) but the lowest for non-dairy milk coffee beverage advertisements (7%). Conversely, while the USA had the second-highest visibility for coffee advertisements (74%), it scored the highest for non-dairy milk advertisements at 65%.

Furthermore, the regions of the UAE and USA appeared to be more conscious of overall calorie intake and the calories in milk, as indicated by higher means on the relevant TDF statements (Table 4). The UK and Germany demonstrated a stronger upbringing on dairy and the belief that it should be part of a diet.

## 4. Discussion

The findings from this study provide a nuanced understanding of consumer behavior and preferences surrounding the use of non-dairy milks in coffee consumption. Taste emerged as one of the primary drivers influencing participants’ coffee choices, highlighting the significance of sensory satisfaction in beverage selection. Many participants indicated a preference for adding cow’s milk to their coffee, highlighting a strong preference for dairy taste, established dietary habits, and potential cultural influences.

Sensory properties are crucial in plant-based drinks, as highlighted by prior research [4,36]. Established consumers of plant-based alternatives generally perceive the taste positively, whereas taste perception remains a significant barrier for non-consumers [37]. Previous studies have shown that expectations and negative associations are common among those who have never tried plant-based beverages, with these associations varying by product type [28]. Additionally, food neophobia—a fear of trying unfamiliar foods—negatively affects the perceptions of plant-based beverages.

A significant aspect of this study was the awareness among the participants regarding extra charges for non-dairy milks at coffee shops. This issue generated diverse opinions, with some arguing against such charges on the grounds of fairness, particularly for individuals with allergies or intolerances for whom plant-based alternatives are the only option. Conversely, supporters of the surcharges cited higher production costs for plant-based alternatives. In reality, one of the primary challenges facing PBMAs is their elevated production costs, which impact every stage of the plant-based milk supply chain [9].

Notably, younger generations like Gen Z and Millennials exhibited stronger opposition to these fees. This inclination could stem from their upbringing in an era marked by extensive access to information and heightened awareness of social and environmental issues, such as the importance of planetary health and the United Nations Sustainable Development Goals. Conversely, older age groups may demonstrate a more nuanced perspective, possibly influenced by evolving societal norms and personal experiences over time. The middle age group, in particular, seems to weigh economic sustainability alongside inclusivity, reflecting a stage of life where financial responsibilities and stability become more prominent concerns. Understanding these generational dynamics is crucial for businesses aiming to align with the values and preferences of their evolving consumer base, ensuring that their practices resonate with both economic realities and social aspirations across different age demographics.

Regional disparities were also evident, with the respondents from the UK and Germany being more inclined to oppose the extra charges for non-dairy milks compared to those from the UAE and USA, where economic factors often tempered inclusivity concerns. This outcome may be influenced by Starbucks’ pricing strategy, which, since 2023, does not impose additional charges for non-dairy milk in the UK and Germany, whereas, in the UAE and USA, there is an extra charge for non-dairy milk in their coffees. Interestingly, despite this inclusive pricing, individuals from the UK and Germany were raised with a stronger emphasis on dairy consumption, often viewing it as a dietary staple more so than their counterparts from the UAE and USA. This difference could be attributed to robust marketing campaigns, the perceived nutritional benefits of dairy milk, or traditional dietary habits and education. It is important to note that a preference for dairy does not undermine the belief in inclusivity, but rather reflects personal choices or perceptions of dairy’s benefits.

This study also revealed nuances in consumer awareness and preferences regarding calorie consciousness in general and in milk products, particularly evident in regions like the USA and UAE. This heightened awareness may explain why coffee shops or brands in these regions target their customers with advertisements for non-dairy milk options, which generally contain fewer calories, based on the respondents’ feedback.

Moreover, while the participants widely recalled advertisements for coffee shops or brands, campaigns specifically promoting non-dairy milk beverages were notably less memorable. This discrepancy suggests that there is significant room for improvement in marketing strategies aimed at increasing the visibility and appeal of plant-based alternatives. Targeted marketing efforts could effectively address this gap by highlighting the benefits of non-dairy milks, such as their environmental sustainability [1,2], health advantages [4,9], and suitability for those with dietary restrictions [7,8,9]. By focusing on these unique selling points, companies could not only enhance consumer awareness but also foster a stronger connection with the growing demographic that values inclusivity and sustainability in their dietary choices.

### Behavior Change Suggestions

The results from the regression analysis showed that the participants who viewed non-dairy milk as a dietary staple were more likely to consume it. This finding aligns with the “Beliefs about Consequences” domain from the Theoretical Domains Framework (TDF) (refer to Table 1 and Appendix A for details).

The Belief about Consequences domain refers to an individual’s perception of the positive or negative outcomes and the effects associated with specific behaviors or actions [22]. The participants who see non-dairy milk as a dietary staple likely believe in its positive outcomes, such as health benefits [4,9], environmental sustainability [1,2], or alignment with dietary restrictions [7,8,9]. These anticipated positive consequences potentially reinforce their choice to consume non-dairy milk. Their consumption behavior is influenced by their belief in the favorable results of integrating non-dairy milk into their diet.

**Figure 5:**
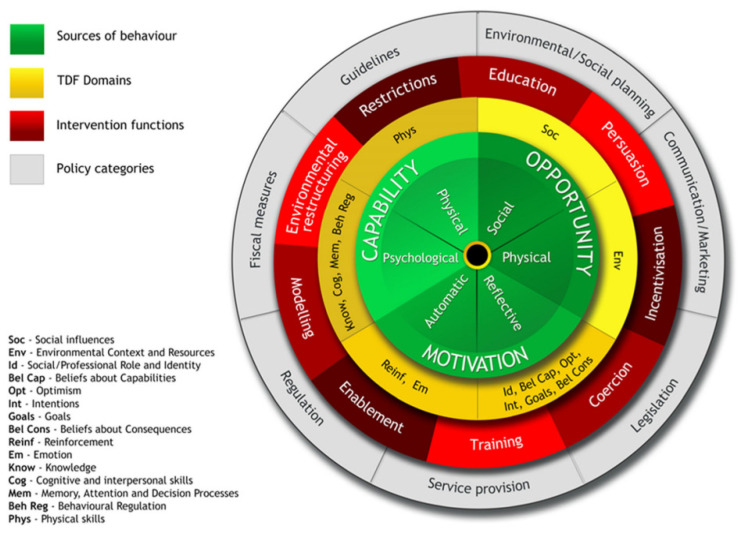
Behavior Change Wheel with TDF Domains mapped [23,38].

The Behavior Change Wheel (BCW) [23] can be used to transition from identifying behavioral patterns to designing interventions. Ojo et al. [38] have adapted the BCW, as shown in Figure 5, to illustrate how the Theoretical Domains Framework (TDF) domains align with the BCW. This mapping facilitates the selection of appropriate intervention functions and policy categories that can effectively address the behavioral determinants identified in our study. In the BCW, the intervention functions corresponding to the Beliefs about Consequences domain are Training and Coercion.

To align with the Training function of the Behavior Change Wheel (BCW), coffee shops can implement comprehensive training programs for baristas that extend beyond the basics of non-dairy milk preparation. These programs can include advanced preparation techniques, where baristas are trained in how to steam and froth various non-dairy milks to achieve the perfect texture and consistency for different coffee beverages. Sensory training is also essential, as educating staff on the taste, mouthfeel, and aroma profiles of different non-dairy milks will allow them to make informed recommendations to customers based on individual taste preferences. This approach can help to address food neophobia and challenge the negative expectations and associations commonly held by those unfamiliar with plant-based beverages [28]. Our survey results indicate that taste is a significant factor in coffee consumption decisions. Additionally, providing baristas with knowledge about the nutritional benefits and potential allergens associated with different non-dairy milks will help them to address customer inquiries more accurately and confidently. Educating staff in the coffee shops on the positive consequences of drinking plant-based alternatives for both individuals and the planet’s health, as well as informing them about sustainability and ethical issues, can further enhance customer engagement and satisfaction.

In terms of Coercion, which involves the expectation of costs or punishment [23], it can be implemented in a more positive and strategic manner to promote non-dairy milk consumption. Offering time-limited discounts or promotions on beverages made with non-dairy milk can be an effective approach. For instance, implementing “Non-Dairy Mondays” where all non-dairy milk beverages are discounted encourages customers to try them out. Developing loyalty programs that reward customers for choosing non-dairy milk is another strategy (similar to the Starbucks “Gold” program). For example, a point system where customers earn extra points for each non-dairy milk purchase, which can be redeemed for free beverages or other rewards, can enhance customer loyalty.

Environmental incentives can also be tied to non-dairy milk consumption. Implementing eco-friendly initiatives where customers who bring reusable cups and choose non-dairy milk receive a discount or a small reward promotes not only non-dairy milk but also sustainable practices. Subscription plans can be introduced for frequent customers, offering benefits such as free non-dairy milk options or reduced surcharges for non-dairy milk over a set period. Special events and campaigns can further promote non-dairy milk. Hosting events like a “Non-Dairy Milk Week” with special offers, tastings, and informational sessions can raise awareness and encourage more customers to try non-dairy milk options.

By incorporating these strategies, both training and coercion can be effectively used to promote the adoption and acceptance of non-dairy milk options in coffee shops. This comprehensive approach will ultimately lead to a more inclusive and health-conscious customer base, while also supporting environmental sustainability.

## 5. Conclusions

The present study examined consumer preferences and behaviors related to non-dairy milk alternatives in the context of coffee consumption. Based on the results of the survey, we gained valuable insights into the demographics, coffee-related responses, dairy milk alternative preferences, marketing, and beliefs that influence consumer choices. This study revealed that taste was the primary reason participants drank coffee. Most participants consumed coffee at home, followed by coffee shops and the office. Cow’s milk was the preferred additive in coffee for the majority of the respondents. Many favored local cafes or small businesses, citing coffee quality or taste as reasons why. A significant number of participants were aware of extra charges for non-dairy milks at coffee shops, with a majority reporting that their coffee shop applied such charges. Arguments against extra charges included concerns that they should not be due to allergies or intolerances, while those in favor argued that production costs for plant-based alternatives were higher. Gen Z and Millennials were particularly likely to oppose charges for non-dairy milks due to lactose intolerance or allergies. The respondents from the UK and Germany were more inclined to believe that no one should pay extra for non-dairy milk compared to those from the UAE and USA, where economic considerations balanced inclusivity concerns. The majority of participants remembered encountering advertisements for coffee shops or brands, while only approximately one-third recalled seeing campaigns promoting non-dairy milk beverages. Finally, the results from the regression analysis indicated that the participants who viewed non-dairy milk as a dietary staple were more likely to consume it. This finding aligns with the “Beliefs about Consequences” domain from the Theoretical Domains Framework (TDF), as these individuals likely associate non-dairy milk with positive outcomes, such as health benefits and environmental sustainability. Finally, based on the BCW, practical suggestions for intervention strategies based on Training and Coercion were discussed.

As with any survey, there are limitations to consider. Although the findings were statistically significant, the sample size of 200 participants does not comprehensively represent the diverse perspectives and preferences of all coffee consumers. A larger sample size would enhance the reliability and generalizability of the results. Conducting more extensive surveys in each of the top five countries, which provided particularly interesting insights, could yield stronger and more nuanced findings. Additionally, future studies should delve deeper into the impact of cultural factors on milk preferences. Cultural norms, dietary habits, and traditional beliefs can significantly influence consumer behavior and attitudes towards dairy and non-dairy milk. Understanding these cultural influences would provide a more holistic view of consumer preferences and could inform more targeted marketing and policy-making efforts. This expanded approach would help us to create a comprehensive understanding of the global market for dairy and non-dairy milk in coffee consumption.

## Figures and Tables

**Table 1:** TDF statement descriptions.

	TDF Items	Min	Max	Mean	SD	Var
1	I drink non-dairy milk. (Beliefs about Capabilities)	1.00	5.00	3.07	1.61	2.61
2	Non-dairy milk tastes bad. (Beliefs about Consequences)	1.00	5.00	2.65	1.28	1.64
3	Non-dairy milk is too expensive. (Environmental Context and Resources)	1.00	5.00	3.35	1.03	1.07
4	Non-dairy milk is not available to me. (Environmental Context and Resources)	1.00	5.00	1.76	1.07	1.15
5	My friends/colleagues/family do not drink non-dairy milk. (Social Influences)	1.00	5.00	2.59	1.31	1.73
6	Dairy milk (cow’s milk) should be part of a normal diet. (Beliefs about Consequences)	1.00	5.00	2.91	1.07	1.15
7	I grew up drinking dairy (cow’s milk). (Social Role and Identity)	1.00	5.00	4.47	0.98	0.95
8	Non-dairy milk should be part of a normal diet. (Beliefs about Consequences)	1.00	5.00	3.16	0.96	0.92
9	I am conscious about the amount of calories I consume. (Behavioral Regulation)	1.00	5.00	3.40	1.24	1.54
10	I am conscious about the amount of calories in different types of milk. (Knowledge, Behavioral Regulation)	1.00	5.00	2.68	1.38	1.91

**Table 2:** Regression analysis results.

**Consumption of Plant-Based Milk Alternatives in the Coffee Drink**				
F (10, 189)	10.462, *p* < 0.001			
Multiple R	0.5969			
R^2^	0.356			
**Independent Variable**	***ß***	***SE***	***t***	*p*-Value *
I drink non-dairy milk.	0.124	0.020	6.121	0.000
Non-dairy milk tastes bad.	−0.023	0.028	−0.822	0.412
Non-dairy milk is too expensive.	−0.030	0.028	−1.041	0.299
Non-dairy milk is not available to me.	−0.023	0.027	−0.836	0.404
My friends/colleagues/family do not drink non-dairy milk.	0.005	0.022	0.244	0.807
Dairy milk (cow’s milk) should be part of a normal diet.	−0.072	0.031	−2.338	0.020 *
I grew up drinking dairy (cow’s milk).	−0.017	0.030	−0.572	0.568
Non-dairy milk should be part of a normal diet.	0.066	0.031	2.177	0.036 *
I am conscious about the amount of calories I consume.	0.015	0.029	0.518	0.605
I am conscious about the amount of calories in different types of milk.	−0.007	0.026	−0.256	0.798

* *p* < 0.05.

**Table 3:** Marketing campaigns awareness frequencies based on location.

**Have You Seen a Coffee Advertisement in the Last 6 Months?**				
	**N**	**Yes**	**No**	**I Do Not Remember**
USA	46	74%	15%	11%
UAE	43	49%	23%	28%
UK	22	55%	9%	36%
Germany	21	67%	10%	24%
Canada	17	82%	12%	6%
**Have You Seen a Non-Dairy Milk Coffee Beverage Advertisement in the Last 6 Months?**				
USA	39	65%	14%	21%
UAE	33	23%	39%	38%
UK	20	40%	15%	45%
Germany	19	42%	32%	26%
Canada	15	7%	67%	27%

**Table 4:** TDF statement means based on location.

Respondents’ Country	Grew Up Drinking Milk		Non-Dairy Should Be Part of a Diet		Dairy Should Be Part of a Diet		Conscious about Overall Calories		Conscious about Milk Calories	
	Mean	Var	Mean	Var	Mean	Var	Mean	Var	Mean	Var
UAE and USA	4.4	0.8	3.4	0.8	3.1	1.0	3.9	1.0	3.3	1.9
UK and Germany	4.8	0.2	2.4	0.8	3.7	2.3	3.2	1.7	2.0	1.8

## Data Availability

Data are contained within the article.

## Appendix A. Survey Questions

**Demographics:**		
1	What is your age?	
2	What gender do you identify as?	
3	What country do you live in (spend most of your days in a year)?	
4	What is your employment status?	
5	If employed, do you work from home, office, or hybrid?	
6	Do you have any food allergies?	
7	Do you drink coffee?	
**Coffee-related behavior:**		
1	Why do you drink coffee?	
2	How often do you drink coffee?	
3	If daily, how many coffee beverages do you consume a day on average?	
4	What time of the day do you drink coffee?	
5	Do you drink your coffee hot or cold?	
6	Where do you drink your coffee?	
7	What kind of milk do you take with your coffee, if any?	
8	Which coffee shop or brand do you prefer the most to drink from?	
9	Why do you prefer to drink from the coffee shop/brand you selected in the previous answer?	
**Attitudes towards non-dairy milk charges at coffee shops:**		
1	Your opinion on dairy milk alternatives: does the coffee shop you visit or are aware of charge for non-dairy milks (i.e., soy, almond, oats, other)?	
2	Should customers pay extra for non-dairy milks (i.e., soy, almond, oats, other)?	
3	If the coffee shop did not charge extra for non-dairy milks, would you visit it/order from it more often?	
**Marketing campaigns awareness:**		
1	In the last 6 months, have you seen any advertisement for coffee/coffee shop (either in public or online/mobile or other)?	
2	Have you seen any advertisement for non-dairy milk coffee beverages in the same period?	
**Theoretical-Domains-Framework-based questions:** How much do you agree with the following statements about non-dairy milk (i.e., soy, almond, oats, other)? (5-point Likert scale)		
	**TDF Statement**	**TDF Domain**
1	I drink non-dairy milk.	Beliefs about Capabilities
2	Non-dairy milk tastes bad.	Beliefs about Consequences
3	Non-dairy milk is too expensive.	Environmental Context and Resources
4	Non-dairy milk is not available to me.	Environmental Context and Resources
5	My friends/colleagues/family do not drink non-dairy milk.	Social Influences
6	Dairy milk (cow’s milk) should be part of a normal diet.	Beliefs about Consequences
7	I grew up drinking dairy milk (cow’s milk).	Social Role and Identity
8	Non-dairy milk should be part of a normal diet.	Beliefs about Consequences
9	I am conscious of the number of calories I consume.	Behavioral Regulation
10	I am conscious of the number of calories in different types of milk.	Knowledge, Behavioral Regulation
